# Microsporidia Intracellular Development Relies on Myc Interaction Network Transcription Factors in the Host

**DOI:** 10.1534/g3.116.029983

**Published:** 2016-07-05

**Authors:** Michael R. Botts, Lianne B. Cohen, Christopher S. Probert, Fengting Wu, Emily R. Troemel

**Affiliations:** Division of Biological Sciences, Section of Cell and Developmental Biology, University of California, San Diego, La Jolla, California 92093

**Keywords:** *C. elegans*, *N. parisii*, intestine, microsporidia, pathogenesis, Genetics of Immunity

## Abstract

Microsporidia are ubiquitous parasites that infect a wide range of animal hosts, and these fungal-related microbes undergo their entire replicative lifecycle inside of host cells. Despite being widespread in the environment and causing medical and agricultural harm, virtually nothing is known about the host factors important to facilitate their growth and development inside of host cells. Here, we perform a genetic screen to identify host transcription factors important for development of the microsporidian pathogen *Nematocida parisii* inside intestinal cells of its natural host, the nematode *Caenorhabditis elegans*. Through this screen, we identified the *C. elegans* Myc family of transcription factors as key host regulators of microsporidia growth and development. The Mad-like transcription factor MDL-1, and the Max-like transcription factors MXL-1 and MXL-2 promote pathogen levels, while the Myc-Mondo-like transcription factor MML-1 inhibits pathogen levels. We used epistasis analysis to show that MDL-1 and MXL-1, which are thought to function as a heterodimer, appear to be acting canonically. In contrast, MXL-2 and MML-1, which are also thought to function as a heterodimer, appear to be acting in separate pathways (noncanonically) in the context of pathogen infection. We also found that both MDL-1::GFP and MML-1::GFP are expressed in intestinal cells during infection. These findings provide novel insight into the host transcription factors that regulate microsporidia development.

Microsporidia comprise a phylum of fungal-like obligate intracellular parasites that infect all phyla of animals, including humans and agriculturally important animals (Vavra and Lukes 2013). Disease caused by microsporidia infection poses a threat in an agricultural setting, particularly in commercial apiculture and aquaculture (Stentiford *et al.* 2013; Stentiford *et al.* 2016; Higes *et al.* 2013; Troemel 2011). In addition, there are 14 species of microsporidia that are known to cause disease in humans, including life-threatening wasting diarrhea in immunocompromised people (Didier and Weiss 2011). Furthermore, recent estimates have found that microsporidia may infect up to 56% of immunocompetent people, and the true impact of microsporidia infection on human health is poorly understood (Sak *et al.* 2011). Despite the prevalence of disease associated with microsporidia infection, there is a dearth of effective treatments for treating microsporidiosis.

Microsporidia have a very specialized obligate intracellular lifestyle, which begins with the transmissible spore form (Figure 1A) (Keeling and Fast 2002). This spore contains a coiled polar tube that can rapidly fire and pierce host cells, and through which a parasite cell called a sporoplasm can be injected directly into a host cell. The sporoplasm develops into an intracellular multinucleate replicative form called a meront, which then differentiates back into spores that are shed by the host. Thus, microsporidia undergo all of their replication inside of the host cell. Microsporidia complete this infectious lifecycle with some of the smallest known eukaryotic genomes [as small as 2.3 Mb in one human-infecting species (Corradi *et al.* 2010)], having lost genes conserved among other eukaryotes (Katinka *et al.* 2001; Texier *et al.* 2010; Williams 2009). For example, microsporidia have lost mitochondrial pathways like oxidative phosphorylation, in keeping with their lack of true mitochondria. Genome compaction in obligate intracellular pathogens such as microsporidia results in dependence on host resources to facilitate intracellular growth, and pathogen hijacking of host cell processes to redirect these resources. One such mechanism by which microsporidia may acquire host resources is through microsporidia-encoded nucleotide and nucleoside transport proteins that import nucleotides and nucleosides from the host-cell cytoplasm into the parasite cell (Tsaousis *et al.* 2008; Cuomo *et al.* 2012). However, almost nothing is known about which host cell machinery is important for providing a hospitable host cell environment for microsporidian growth.

**Figure 1:**
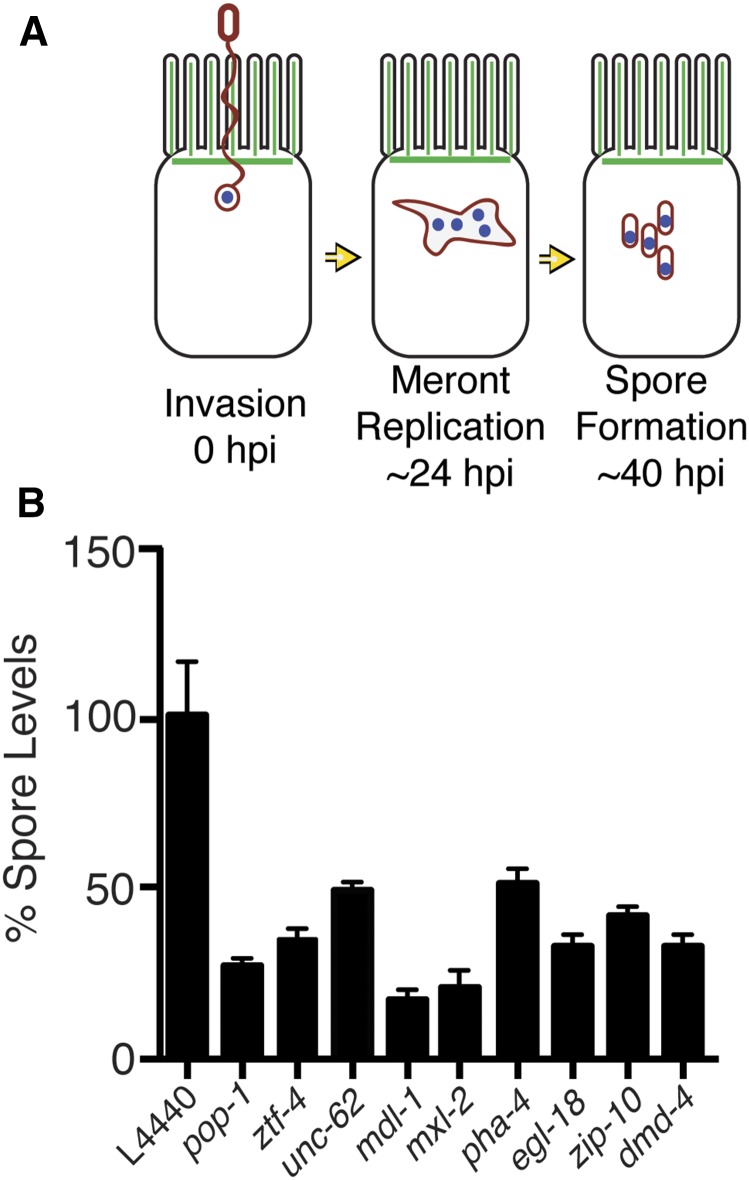
RNAi clones identified in transcription factor library screen cause decreased spore levels in infected animals. (A) After *N. parisii* invades *C. elegans* intestinal cells, it replicates in a meront form, and then differentiates into spores. (B) Spore levels (spores/animal) of infected N2 animals after treatment with RNAi, using clones that were hits from the screen (Table S1). Spore levels were measured with DY96 staining of spores and quantification by hemocytometer. All RNAi treatments were normalized with a L4440 empty vector control, which was set to a baseline of 100% spore levels. Spore levels shown as the mean of three independent experiments. Error bars are SEM. All RNAi conditions were significantly different than empty vector treated animals (*P* < 0.0001).

Several studies have identified changes in gene expression that occur during microsporidia infection, and have identified conserved pathways that are regulated by infection (Szumowski and Troemel 2015). These pathways include the ubiquitin proteasome system (UPS) in *Caenorhabditis elegans* infected with *Nematocida parisii* (Bakowski *et al.* 2014), and the type-I interferon response pathways in human foreskin fibroblasts infected with *Anncallia algerae* (Panek *et al.* 2014). However, the functional consequences of these gene expression changes are not well understood, and no transcription factors have been identified that are responsible for controlling these changes. Indeed, virtually nothing is known about host factors that aid in growth of microsporidian pathogens in any system.

Here, we describe the use of *N. parisii*, a natural microsporidian pathogen of *C. elegans* nematodes (Troemel *et al.* 2008), for high throughput RNAi screening to identify host transcription factors that are important for pathogen development in a whole animal. We identified several conserved transcription factors required for normal pathogen development, including components of the *C. elegans* Myc interaction network, which is reduced in number compared to other animals (Pickett *et al.* 2007; Johnson *et al.* 2014). In mammals, the Myc transcription factor was originally identified for its role in cancer, and it has several interaction partners that, together with Myc, have been shown to regulate gene expression, cell growth, differentiation, apoptosis, and energy metabolism (Tu *et al.* 2015). Orthologs for the mammalian Myc interaction network of transcription factors have been identified in *C. elegans*, and there are four major players in this network (*mdl-1*, *mxl-1*, *mxl-2*, and *mml-1*) that have been shown to regulate tissue differentiation, metabolism, and animal lifespan (Johnson *et al.* 2014; Riesen *et al.* 2014; McFerrin and Atchley 2011; Pickett *et al.* 2007; Nakamura *et al.* 2016). We found that all of these transcription factors regulate *N. parisii* pathogen levels, acting later during the microsporidia life cycle to affect spore levels. Three of these factors (*mdl-1*, *mxl-1*, and *mxl-2)* promote pathogen development, while one of them (*mml-1*) represses pathogen development. Our epistasis analysis indicates there is both canonical and noncanonical activity of these transcription factors during infection. We also analyze ModENCODE data for transcription factors binding sites in the promoter regions of infection-regulated genes. In addition, we show that MDL-1::GFP and MML-1::GFP proteins are expressed in intestinal nuclei during infection, and we characterize other transcription factors that regulate pathogen development for their interaction with MDL-1, identifying new genetic interactions for this factor. Altogether, these findings describe results of the first genetic screen for host factors important for establishing a replicative niche for microsporidia, and identify novel targets that could be used to combat microsporidia-associated disease.

## Materials and Methods

### C. elegans strains

*C. elegans* was maintained on nematode growth media (NGM) seeded with *Escherichia coli* OP50-1 as described (Brenner 1974). Previously generated strains used in this study include:

- N2
- OP106 *unc-119(ed3) III*; *wgIs106 [mdl-1*::*TY1*::*EGFP*::*3xFLAG + unc-119(+)]*
- OP527 *unc-119(tm4063) III*; *wgIs527[mml-1*::*TY1*::*EGFP*::*3xFLAG + unc-119(+)]*
- CB4037glp-1(e2141) III

*mdl-1(tm311)*, *mxl-1(tm1530)*, *mxl-2(tm1516)*, and *mml-1(ok849)* mutants were each backcrossed to N2 wild type to generate these strains. The *mdl-1(tm311) and mxl-2(tm1516)* mutants were obtained from the National Bioresource Project of Japan and the *Caenorhabditis* Genetics Center, and backcrossed in our lab to N2 three times. The *mxl-1(tm1530)* and *mml-1(ok849)* mutants were a kind gift from Dr. Andrew Samuelson, and were backcrossed to N2 three times in their lab, and then backcrossed a further three times into N2 in our lab, because preliminary results indicated there were differences in N2 backgrounds contributing to pathogen growth. These backcrossings led to these four strains:

- ERT414 *mdl-1(tm311*) X
- ERT480 *mxl-1(tm1530)* V
- ERT353 *mxl-2(tm1516)* III
- ERT355 *mml-1(ok849)* III

Double mutants were constructed by backcrossing *mxl-1(tm1530)* or *mxl-2(tm1516)* males with *mdl-1(tm311)* hermaphrodites:

- ERT418 *mxl-1(tm1530) V*;*mdl-1(tm311) X*
- ERT419 *mxl-2(tm1516) III;mdl-1(tm311) X*

GFP transgene complementation lines were made by crossing ERT414 *mdl-1(tm311) X* with OP106 *wgIs106 [MDL-1*::*EGFP*::*TY1*::*3xFLAG]* to generate ERT447 *mdl-1(tm311) X*; *wgIs106*, and by crossing *mml-1(ok849)* with OP527 *wgIs527 [MML-1*::*EGFP*::*TY1*::*3XFLAG]* to generate ERT420 *mml-1(ok849) III*; *wgIs527*. Oligonucleotides used to PCR genotype cross progeny are as follows: *mdl-1(tm311)* forward TCG GTG CTT TCC TAG TTC GT, reverse ATT GGA CCC CTT GGG ATA AG; *mxl-1(tm1530)* forward GGG GAC AAG TTT GTA CAA AAA AGC AGG CTG GAT GTC TGA CAT GAG TGA CCT CG, reverse GGG GAC CAC TTT GTA CAA GAA AGC TGG GTG TTG AAT TAT CGA CTG CGA TG; *mxl-2(tm1516)* forward GCC AGC TCC TCT CAA AAG CCG, reverse CTG TCG ACT TGA ATG GCT CCG C; and *mml-1(ok849)* forward CGA GTC CAC CTG TGA ACT GG, reverse GAG AAC TCC GAC GAA TGA TCA GCC.

### Spore levels RNAi screen

To identify RNAi clones that cause a decrease in spore levels, we developed a screen to quantify spore levels in animals grown in 96-well plates. RNAi bacteria were grown in 1 ml of Luria Broth (LB) containing 50 µg/ml carbenicillin in deep-well 96-well plates overnight with shaking. The bacteria were then subjected to centrifugation at 5000 × *g* for 15 min and the supernatant discarded. The bacteria were then resuspended in 100 µL of S-medium containing 1 mM isopropyl β-d-1-thiogalactopyranoside (IPTG). Synchronized *glp-1(e2141)* L1 animals were suspended in S-medium containing IPTG, and then dispensed in 96-well plates in a 50 µl volume with 200 animals per well. Fifteen microliters of RNAi bacteria were then added to each well, and the plates were incubated at 25° with shaking at 200 rpm in the dark. After 24 hr, 1 × 10^6^ spores were added to each well in 10 µl of S-Medium with 1 mM IPTG along with an additional 15 µl of RNAi bacteria. At 24 hr post-inoculation (hpi), an additional 25 µl of RNAi bacteria was added to each well to prevent starvation. At 44 hpi 15 µl of 16% paraformaldehyde was added to each well, and plates were placed at 4°.

Infected animals were then transferred to deep-well 96-well plates, and washed three times with phosphate buffered saline (PBS) containing 1% sodium dodecyl sulfate (SDS). A staining solution of 5 µg/mL Direct Yellow 96 (DY96) in PBS with 1% SDS was added to each well, and the plates were incubated at room temperature overnight. The staining solution was washed with five washes with PBS plus 1% SDS (5 min room temperature incubation between each wash), followed by two washes with PBS with 0.1% Tween-20. Stained animals were then transferred to clear-bottom 96-well plates, and fluorescence was quantified using a BMG Labtech Novostar plate reader.

### Spore levels assay

Spore levels were quantified as described (Szumowski *et al.* 2014). Briefly, 500 synchronized N2 L1s were grown on RNAi plates for 24 hr at 25°. These animals were then washed off plates using M9, and transferred to a fresh 6-cm RNAi plate seeded with RNAi bacteria and 2 × 10^6^ *N. parisii* spores, then grown at 25°. At 40 hpi, infected adult animals were washed off of the plates using M9, and then fixed in acetone. Fixed animals were washed three times with PBS containing 0.1% Tween-20 (PBS-T), before transferring to a 96-well plate for sorting with the Union Biometrica COPAS Biosort machine to dispense 50 animals per well into a new 96-well plate. Wells containing 50 infected animals in 50 µl of water were then mixed with 150 µl of lysis buffer containing PBS with 2% SDS, 0.01% 2-mercaptoethanol, and 4 µg/ml DY96; lysis was monitored microscopically, and complete lysis was typically achieved after 2 hr incubation at room temperature. Spores were then counted by visualizing DY96 stained spores on a hemocytometer.

Spore levels were quantified in mutant worms identically as described above, except that RNAi bacteria with an empty RNAi vector was used (HT115 *E. coli* with L4440 plasmid) as the food source. All experiments were performed in independent triplicates and analyzed for statistical significance using ANOVA and GraphPad software (Prism).

### Sporoplasm counting assay

*N. parisii* sporoplasms were quantified as described (Balla *et al.* 2015). Briefly, synchronized L1s were added to RNAi plates seeded with a mixture of HT115 *E. coli* and 4 × 10^6^ *N. parisii* spores. Animals were allowed to feed on the bacteria and spore mixture for 4 hr at 25° before fixing with 4% paraformaldehyde on ice for 30 min. Fixed animals were then washed with PBS-T prior to staining with MicroB fluorescent in situ hybridization (FISH) probe against *N. parisii* rRNA. Sporoplasms within intestinal tissue were counted microscopically using a Zeiss AxioImager microscope and a 40 × objective. At least 100 animals were counted for each replicate, with three independent experiments performed. Mean number of sporoplasms per worm was compared using ANOVA and GraphPad software (PRISM).

### Pathogen load analysis by FISH

Pathogen load was analyzed as described (Balla *et al.* 2015). Briefly, animals were washed off infection plates, fixed with acetone and then stained with a mix of five oligonucleotide probes, named Micro A–E conjugated to the red Cal Fluor 610 dye (Biosearch Technologies) that are specific to *N. parisii* ribosomal RNA. Pathogen load was measured with the COPAS Biosort (Union Biometrica) by quantifying red fluorescence, and normalizing to time of flight to measure size. Events were gated to remove events that were not of worm size.

### Identification of transcription factor binding sites near N. parisii infection-regulated genes

To identify transcription factor binding sites, we downloaded modENCODE high confidence peak transcription factor binding sites (TFBS) data, using the “TF binding site” file for 22 transcription factors reported in Gerstein *et al.* (2010), using modENCODE release #33 (http://intermine.modencode.org/release-33/begin.do). We then used bedTools version 2.22.1 to derive the [–2000 bp, +150 bp] regions of genes in the WB190 *C. elegans* genome to perform an analysis of the intersection between these regions and the high confidence modENCODE TFBS regions. We then took a previously described RNA-seq dataset from a time-course of *C. elegans* infected with *N. parisii* (Bakowski *et al.* 2014), found the number of binding sites for each transcription factor within infection-regulated (IR) genes, and used Fisher’s exact test to determine the association of binding sites for each transcription factor with the promoters of IR genes, to determine the odds ratio that a particular transcription factor is enriched for having binding sites in IR genes. We used a two-sided alternative hypothesis, and counted only unique transcription factor-gene upstream sequence intersections (upstream sequences with multiple TFBS were only counted once). We then performed FDR correction for multiple comparisons using the Benjamini-Hochberg procedure with an FDR threshold of 0.01.

### qRT-PCR analysis

RNA was isolated from animals with Tri-Reagent and bromochloropropane (Molecular Research Center). mRNA was reverse transcribed into cDNA using oligo dT and the RETROscript kit (Ambion). cDNA for each sample was quantified with iQ SYBR Green Supermix (Bio-Rad) on a CFx Connect Real-time PCR Detection System (Bio-Rad). *snb-1* was used as a control gene. C17H1.6 was used as a positive control for a gene that is differentially expressed after infection. *mdl-1* forward primer: GGG AGC ACT TAC CTC CAC AT. *mml-1* forward primer: CATTGGAAGCAGAGGGTACG. *mml-1* reverse primer: GTTGTGTCATCGTCGGGTAC. GFP forward primer: TGGCAGACAAACAAAAGAATG. GFP reverse primer: GTTGAACGCTTCCATCTTCA. *snb-1* forward primer: CCG GAT AAG ACC ATC TTG ACG. *snb-1* reverse primer: GAC GAC TTC ATC AAC CTG AGC. *dod-3* forward primer: AAGCCATGTGCATATTGTGG. *dod-3* reverse primer: ATACTGGATCGTCTCAAGTTCG. *elo-6* forward primer: CACCCACTTAAAACCCCACT. *elo-6* reverse primer: AAATAGCAAGACCCGCATTC. *pho-11* forward primer: TCCACAGAAACACGTCAACA. *pho-11* reverse primer: TGTTCGAATTTTCTGGTTTCTC. *clec-53* forward primer: ACTGGAATGATGATGTTCGTTT. *clec-53* reverse primer: GCCATTTTGAGTTTTGATAGACC. Y51F10.7 forward primer: GCGATTATTGACTTGATTGCTG. Y51F10.7 reverse primer: TACTTGACAAACGCCGAACT. Y105C5A.13 forward primer: GGAAATGCGAATCCCAAC. Y105C5A.13 reverse primer: TCTGCACTTGGTTGATCTGAA. Y41C4A.11 forward primer: GATTGCTCAATTTGTGTTTGG. Y41C4A.11 reverse primer: AAAGTTAAGCGTGGTGTTTCC. Fold change was calculated using the Pfaffl method (Pfaffl 2001).

### Western blots

Animals were washed off NGM plates with M9 buffer, and then washed once with M9 to remove bacteria. Samples were then resuspended in sample buffer with 1% SDS and 50 mM dithiothreitol (DTT) and boiled at 95° for 10 min. Lysates were then run on a 4–20% gradient SDS-PAGE gel (Bio-Rad) and transferred to Polyvinylidene fluoride (PVDF) membrane (Bio-Rad). The blots were stained with M2 anti-FLAG antibody (Sigma-Aldrich) diluted at 1:1000 and JLA20 anti-actin antibody (Millipore) diluted at 1:1000. The blots were treated with ECL reagent (Amersham GE Healthcare Life Sciences), and imaged either on a Bio-Rad ChemiDoc or on film.

### Fluorescence imaging of C. elegans

MDL-1::GFP::3xFLAG and MML-1::GFP::3xFLAG-expressing animals were imaged with a Zeiss LSM700 confocal microscope, as previously described (Dunbar *et al.* 2012). All other fluorescence images were captured with a Zeiss AxioImager M1 fluorescence microscope.

### Statistical analysis

All statistical analysis was performed using GraphPad version 6.0 (Prism) software. For comparing multiple means (sporoplasm counting assay, spore levels assay, and FISH pathogen load), one-way ANOVA was performed comparing all means to a wild type control. For all plots, statistical significance is denoted using asterisks (* *P* < 0.05, ** *P* < 0.01, *** *P* < 0.001, **** *P* < 0.0001).

### Data availability

The authors state that all data necessary for confirming the conclusions presented in the article are represented fully within the article.

## Results

### Identification of C. elegans transcription factors that regulate N. parisii spore levels

To identify host genes that are important for the development of microsporidia, we developed a fluorescence plate-reader-based assay to measure the levels of *N. parisii* spores inside of the *C. elegans* host. We used a chitin-binding dye called Direct Yellow 96 (DY96) to label the chitin-containing spore coat of *N. parisii* spores. DY96 fluoresces very brightly in the green channel, which is compatible with most fluorescence plate-readers (Hoch *et al.* 2005). (The most commonly used chitin-binding dye, Calcofluor White, emits in the blue channel, which is less useful for plate reader assays). We stained animals at 44 hpi, when animals are typically filled with > 20,000 spores, thus providing robust DY96 signal (Supplemental Material, Figure S1). However, because DY96 can also stain *C. elegans* eggs (which contain chitin), we used conditionally sterile *glp-1(e2141)* animals and carried out the assay at the nonpermissive temperature of 25° where these mutants do not produce eggs (Priess *et al.* 1987).

With the DY96 plate-based assay, we screened a feeding RNAi library composed of ∼345 *C. elegans* predicted transcription factors to identify factors that regulate pathogen growth. This library represents roughly one-third of the total number of transcription factors in the *C. elegans* genome, and was selected because it has been previously used to identify regulators of pathogen response genes (Estes *et al.* 2010). To screen the library, synchronized *glp-1(e2141)* L1 larvae were grown in liquid culture 96-well plates, and fed bacteria expressing dsRNA against *C. elegans* genes for 24 hr. These animals were then infected with *N. parisii* spores, and 44 hpi they were fixed and stained with DY96 to label newly made spores. Fluorescence intensity was then quantified for each well using a fluorescent plate reader and normalized to the RNAi empty vector control (L4440) well. We used an arbitrary cut-off of < 65% of normalized DY96 fluorescence signal to define RNAi hits that decrease spore levels (Table S1), which identified 32 RNAi clones. Ten clones were discarded because they caused larval arrest or lethal phenotypes, and the remaining 22 clones were tested with a secondary screening assay. To verify the phenotypes, we used a previously described spore levels assay in which infected animals are lysed to release spores, which are then counted using a hemocytometer (Szumowski *et al.* 2014). Nine RNAi clones caused a significant decrease in spore levels at 40 hpi, including two components of the “Myc interaction network”, Mad-Like 1 (*mdl-1)*, Max-Like 2, (*mxl-2)*, and also a FoxA transcription factor, (*pha-4)*, which has been shown to have largely overlapping target genes with *mdl-1* (Figure 1B) (Johnson *et al.* 2014). The identification of three genes related to the Myc interaction network of transcription factors in *C. elegans* suggested that this pathway may be important for regulating *N. parisii* growth, which we further examined as described below.

### C. elegans Myc interaction network controls pathogen levels later in the pathogen life cycle

**Figure 2:**
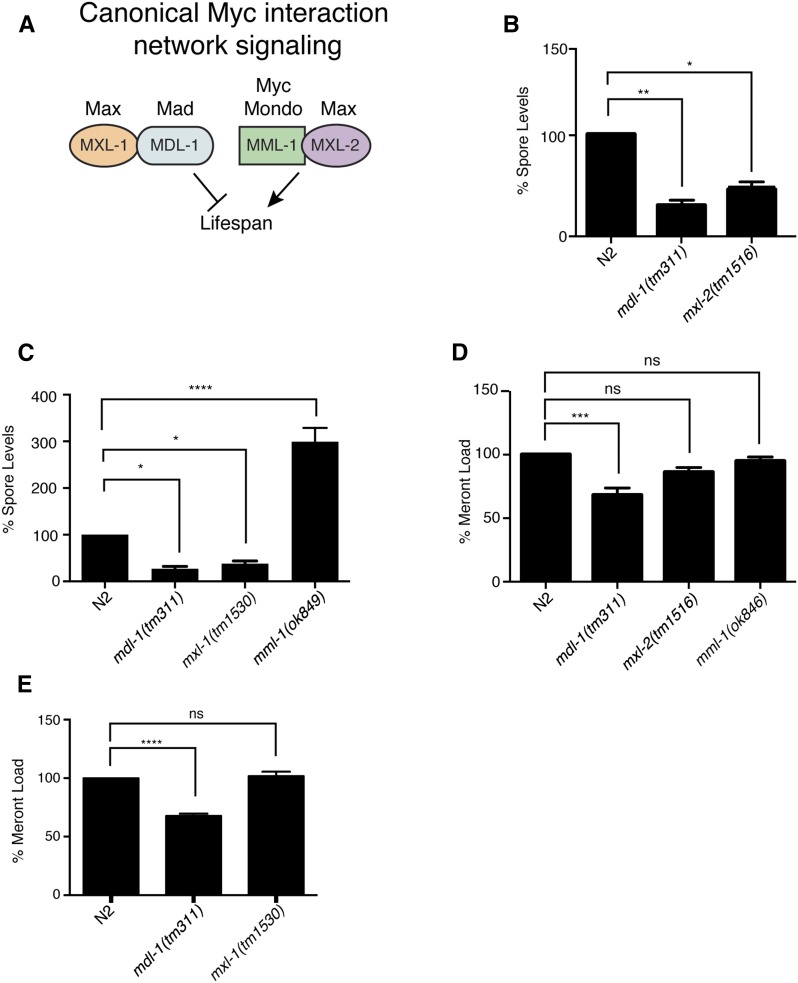
C.*elegans* Myc interaction network transcription factors regulate *N. parisii* pathogen development. (A) Canonical wiring of Myc interaction network transcription factors. The mammalian homologs are listed above the *C. elegans* genes. MML-1 and MXL-2 are thought to act as a heterodimeric pair that promotes transcription and lifespan, and their activity is opposed by MDL-1 and MXL-1 acting as heterodimeric pair. (B and C) Spore levels at 40 hpi shown as the mean of three independent experiments, normalized to wild type animals. (D and E) Pathogen load of *N. parisii* meronts at 24 hpi, measured using an *N. parisii*-specific rRNA FISH probe. Three technical replicates were performed in each of three independent experiments. Fluorescence was measured with a COPAS Biosort and normalized to time-of-flight as a measure of body size, and then normalized to wild-type animals, with at least 325 animals per technical replicate. ns, not significant. * *P* < 0.05, ** *P* < 0.01, *** *P* < 0.001, **** *P* < 0.0001.

Canonical signaling of the factors in the *C. elegans* Myc interaction network, MDL-1, MXL-1, MML-1, and MXL-2 is shown in Figure 2A. To confirm the role of *C. elegans* MDL-1 during *N. parisii* infection, we investigated whether a deletion in *mdl-1* would recapitulate the *mdl-1* RNAi phenotype. *mdl-1(tm311)* mutants have a 500 bp deletion in the *mdl-1* coding region that should lead to a truncated protein of only 64 amino acids (aa) lacking any functional domains, and is thus a presumed null mutation. Indeed, we found that *mdl-1(tm311)* mutants have a dramatic reduction in spore levels at 40 hpi (Figure 2B), confirming this hit from our screen. *C. elegans* MDL-1 protein has been shown to physically interact with the transcription factor Max-like 1 MXL-1 protein in two-hybrid studies (Pickett *et al.* 2007; Grove *et al.* 2009), and studies of aging and proteostasis in *C. elegans* support the model that MDL-1 and MXL-1 act together as a heterodimeric transcription factor (Figure 2A) (Johnson *et al.* 2014). Furthermore, in mammalian systems, the homologs of MDL-1 and MXL-1 (Mad and Max) act as a heterodimeric transcription factor. It was not possible to identify *mxl-1* in our screen because the *mxl-1* RNAi clone was not present in the transcription factor RNAi library we used. Therefore, we investigated its role in spore levels with an *mxl-1(tm1530)* mutant, which contains a 423 bp deletion. Here we found significantly reduced spore levels in *mxl-1(tm1530)* animals (Figure 2C), suggesting that MXL-1 could act together with MDL-1 in a canonical heterodimer to promote *N. parisii* spore levels.

Next, we examined how spore levels are regulated by two other components of the Myc transcription factor network, the Myc-Mondo-like 1 transcription factor (*mml-1)* and the Max-like transcription factor 2 (*mxl-2)*. Based on mammalian Myc data and results in *C. elegans*, it is thought that the MDL-1/MXL-1 heterodimer represses expression of genes activated by a heterodimeric transcription factor composed of MML-1/MXL-2 (Figure 2A) (Johnson *et al.* 2014; Pickett *et al.* 2007; Grove *et al.* 2009). Thus, animals defective in *mml-1* and *mxl-2* would be predicted to have increased spore levels. In support of this model, we found that *mml-1(ok849*)—a mutant containing a 1390 bp deletion in the *mml-1* coding sequence—had higher spore levels compared to wild type animals (Figure 2C). Somewhat surprisingly, however, we found, in a secondary assay from our RNAi screen, that RNAi against *mxl-2* had a phenotype of reduced spore levels (Figure 1B). To further examine this phenotype, we analyzed *mxl-2(tm1516)* mutants, which have a 645 bp deletion that removes the putative DNA-binding domain, resulting in a 64 aa truncated protein with no functional domains, and has been previously characterized to be a null mutant (Pickett *et al.* 2007). Indeed, we found that *mxl-2(tm1516)* mutants had reduced spore levels (Figure 2B), confirming our RNAi results. Thus, there appears to be noncanonical activity of the *C. elegans* Myc interaction transcription factor network during *N. parisii* infection (see epistasis results described in next section for further analysis).

To determine the pathogen growth stage at which the Myc interaction network regulates *N. parisii* development inside of *C. elegans* intestinal cells, we examined pathogen levels at an earlier stage of infection, prior to spore formation. Like all microsporidia, *N. parisii* first replicates in the meront stage before differentiating into spores (Figure 1A). *N. parisii* meronts are actively replicating and spreading throughout the *C. elegans* intestine at the 24 hpi timepoint, and so we quantified pathogen load at this stage using a FISH probe specific to *N. parisii* rRNA to label meronts in N2 wild type, and in *mdl-1(tm311)*, *mxl-1(tm1530)*, *mxl-2(tm1516)*, and *mml-1(ok849)* mutants (Figure 2, D and E). With this assay, *mdl-1(tm311)* animals had a ∼25% decrease in meront levels, while the other mutants did not have a significant change. Thus, MDL-1 may have an effect on pathogen growth at this earlier time point of infection independent of the rest of the Myc interaction network. Overall, these data suggest that the Myc interaction network is more important for pathogen development during the later part of the life cycle when spores are being produced.

Because *mdl-1* mutants have a small but significant effect on pathogen growth at a stage before spore levels, we examined whether this phenotype may be due to a feeding defect that causes these animals to ingest fewer *N. parisii* spores. To determine whether N2 and mutant animals were feeding at a similar rate, and thus receiving similar inoculums of *N. parisii* spores, we counted sporoplasms in wild type and mutant L1 animals after being fed spores for 4 hr. As a positive control to confirm that we can see a feeding difference in this assay, we used *eat-2(ad465)* mutants, which have a known feeding defect. Consistent with *eat-2* mutants having an ∼twofold reduction in feeding, we found that they also have a twofold decrease in the number of sporoplasms/animal. All of the loss of function mutants in the Myc transcription factor family had similar numbers of sporoplasms compared to wild-type animals, and thus do not affect the initial pathogen invasion events into intestinal cells (Figure S2).

### Epistasis analysis of Myc interaction network shows noncanonical roles during infection

**Figure 3:**
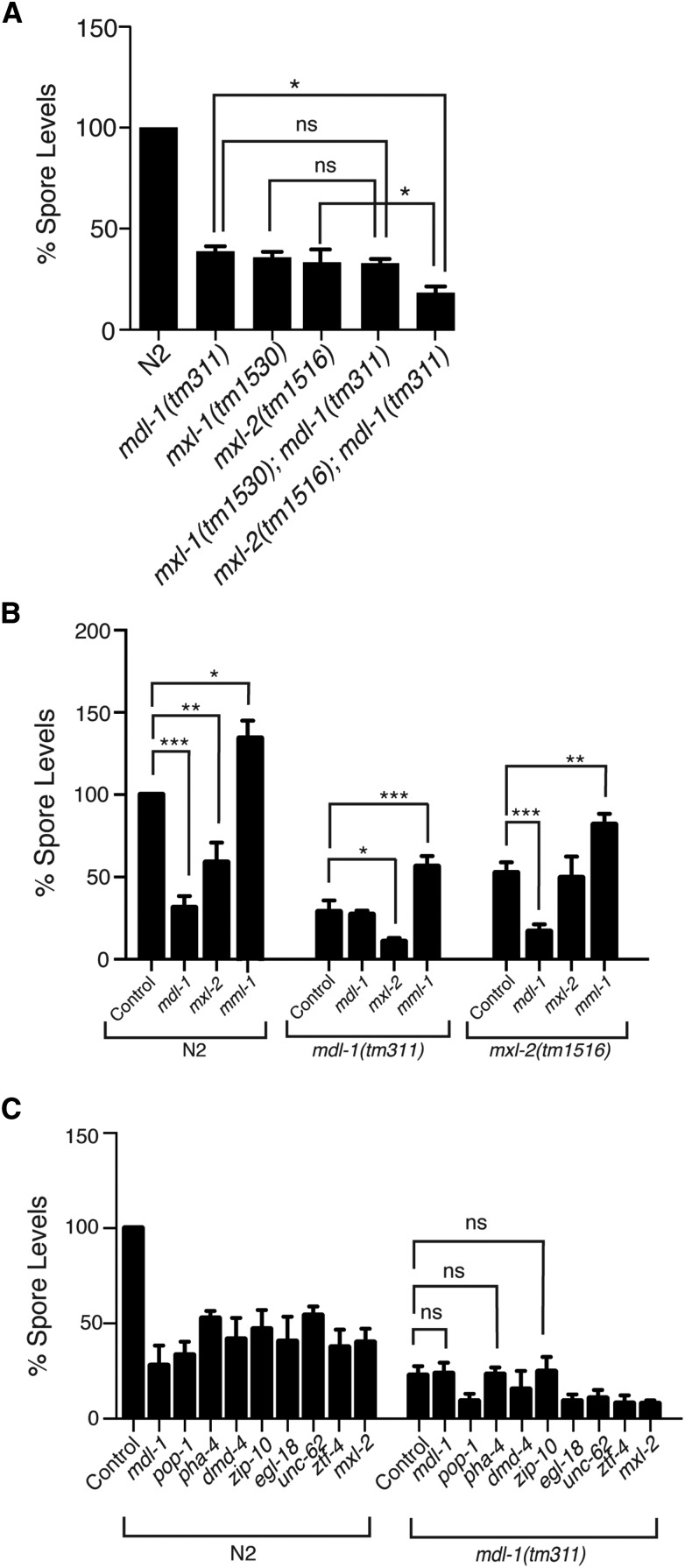
Epistasis analysis of MDL-1, MXL-1, MML-1, and MXL-2 shows noncanonical activity of this pathway during infection. (A) Spore levels at 40 hpi were quantified in N2, *mdl-1(tm311)*, *mxl-1(tm1530)*, *mxl-2(tm1516)*, *mxl-1(tm1530)*;*mdl-1(tm311)*, and *mxl-2(tm1516);mdl-1(tm311) C. elegans*. Spore levels are the mean of three independent experiments, normalized to N2. Error bars are SEM. (B) Spore level values for N2, *mdl-1(tm311)* and *mxl-2(tm1516)* mutants treated with RNAi to knockdown *mdl-1*, *mxl-2*, or *mml-1*. Animal genotypes are denoted by brackets, and each column shows either empty vector control (L4440) or RNAi treatment. (C) Spore levels for N2 and *mdl-1(tm311)* animals treated with RNAi against all other transcription factor library hits (all comparisons with empty RNAi vector control found to be significant except for the three marked “ns”). Error bars represent the SEM for three independent experiments consisting of three replicates per experiment. ns, not significant. * *P* < 0.05, ** *P* < 0.01, *** *P* < 0.001.

To more directly investigate the interactions among components of the Myc interaction network during infection with *N. parisii*, we performed genetic epistasis analysis. First, we investigated interactions between *mdl-1* and other components in the pathway that cause a decrease in spore levels. Canonically, *mdl-1* interacts with *mxl-1* to regulate downstream outputs (Figure 2A) and indeed we found that *mdl-1(tm311);mxl-1(tm1530)* double mutants had the same level of spore reduction as *mdl-1(tm311)* or *mxl-1(tm1530)* single mutants (Figure 3A). This result is consistent with these factors acting together as a heterodimeric complex to regulate gene expression. In contrast, we found that *mxl-2(tm1516);mdl-1(tm311)* double mutants had reduced spore levels compared to either single mutant, indicating that these two factors likely act in parallel (Figure 3A).

Next, we examined the epistatic interactions among these factors with RNAi knock-down in different mutant backgrounds. Consistent with the mutant analysis above, we found that RNAi against *mxl-2* in an *mdl-1* mutant background caused a further decrease in spore levels. We found that RNAi against *mdl-1* in an *mxl-2* mutant background caused a further decrease in spore levels. Thus, based on double mutant analysis, as well as RNAi in both single mutant backgrounds, we find that wild-type *mdl-1* acts in parallel to wild-type *mxl-2* to increase spore levels. We also found that RNAi against *mml-1* still caused an increase in spore levels in an *mdl-1* mutant (Figure 3B), indicating that *mml-1* acts in parallel to *mdl-1*. Similarly, RNAi against *mml-1* still caused an increase in spore levels in an *mxl-2(tm1516)* mutant background (Figure 3B). In summary, we have found that all four major components in the Myc interaction network regulate spore levels, with three components promoting spore levels (*mdl-1*, *mxl-1*, and *mxl-2*), and one component inhibiting spore levels (*mml-1*). *mdl-1* and *mxl-1* appear to act together, while *mxl-2* and *mml-1* act independently of them, and each other.

### Interaction between MDL-1 and other transcription factors that regulate spore levels

One of the other hits from our screen was the FoxA transcription factor PHA-4. Although PHA-4 is not a primary member of the Myc interaction network, previous studies have indicated that there is a significant overlap in genes that contain both a MDL-1 and PHA-4 binding site (Johnson *et al.* 2014). Lifespan studies have shown that the effect of MDL-1 increase in lifespan is dependent on PHA-4. Because of this link, we probed RNAi against PHA-4 and the other transcription factor library hits in an *mdl-1(tm311)* mutant background, which would allow us to detect other transcription factors that may be working in the same pathway as *mdl-1*. When RNAi against the nine screen hits was combined with a loss of *mdl-1* function, there was an additive decrease in spore levels for some genes (*pop-1*, *unc-62*, *egl-18*, *ztf-4*, *dmd-4*, and *mxl-2*), but not for *pha-4*, consistent with the link between *pha-4* and *mdl-1* previously reported (Johnson *et al.* 2014). In addition, there was no further decrease with RNAi against *zip-10*, which suggests that MDL-1 and ZIP-10 may act together—a previously unknown interaction (Figure 3C). Altogether, these data suggest that PHA-4 and ZIP-10 act in the same pathway as the Mad-like transcription factor MDL-1 to regulate *N. parisii* spore levels.

### Analysis of transcription factor regulation of infection response gene expression

One potential role for host transcription factors that regulate spore levels is that they could be mediating a transcriptional response to *N. parisii* infection. Alternatively, these factors could be regulating gene expression independent of infection, and this basal regulation is important for pathogen development. To identify transcription factors that might directly regulate expression of infection response genes, either through induced or basal expression, we compared a modENCODE ChIP-seq dataset (Gerstein *et al.* 2010) with an RNAseq dataset of the *C. elegans* response to *N. parisii* infection that we previously generated (Bakowski *et al.* 2014). We searched for high-confidence transcription factor binding sites found within the promoter regions (–2000 bp and +150 bp from predicted transcription start sites) of infection-regulated genes (genes that are >twofold up or down regulated). We then analyzed these data for: 1) the transcription factor that had binding sites in upstream regions of the greatest number of infection-regulated genes, and 2) enrichment of binding sites for a particular transcription factor in the upstream regions of infection-regulated genes compared to upstream regions of all genes in the genome. We found that MDL-1 had the third highest number of predicted binding sites among the 22 transcription factors from modENCODE that we analyzed (Table S2). However, MDL-1 did not show enrichment for binding sites upstream of infection-regulated genes compared to the rest of the genome (enrichment is defined as an odds ratio > 1, see Table S2). MDL-1 binding sites were only found in 13% of genes (78 of 620 genes) differentially expressed upon infection at any timepoint, while MDL-1 binding sites can be found in 22% of upstream regions of all genes in the genome. We also did not find MDL-1 enriched in the upstream regions of genes upregulated or downregulated by infection when we analyzed these gene sets separately (Table S2). Due to the limited number of transcription factors analyzed in the ModENCODE project, it is difficult to say which transcription factor is the predominant transcription factor that regulates infection response genes in *C. elegans*, but these data suggest that MDL-1 is not the predominant transcription factor that regulates expression of infection-regulated genes.

**Figure 4:**
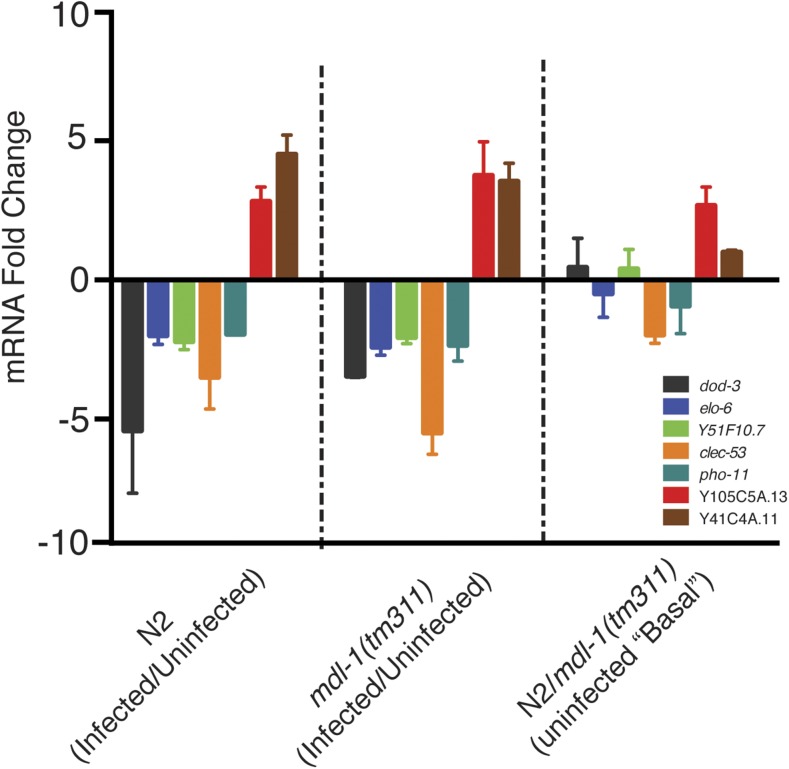
qRT-PCR analysis of the role of MDL-1 in expression of infection-regulated genes. qRT-PCR analysis was performed on infection-regulated genes that have MDL-1 binding sites in their promoter regions, including genes downregulated by infection (*dod-3*, *elo-6*, Y51F10.7, *clec-53*, and *pho-11*), and genes upregulated by infection (Y105C5A.13 and Y41C4A.11). (See Table S3 for RNAseq data.) mRNA fold change is the mean of three independent experiments each consisting of technical duplicates. Error bars are SEM.

Because MDL-1 had such a strong phenotype in controlling pathogen development, however, we further investigated whether it might be directly controlling gene expression of at least some of the infection response genes. Therefore, we next used qRT-PCR to analyze a subset of genes whose transcripts change in response to *N. parisii*, and whose promoters contains a MDL-1 binding site to determine whether MDL-1 was required for their change in expression upon *N. parisii* infection, or for controlling their expression basally. In choosing infection-regulated genes with MDL-1 binding sites we used a more stringent promoter region cut-off of –700 to +100 bp from the transcriptional start site, and analyzed five genes that are repressed during infection (*dod-3*, *elo-6*, *pho-11*, *clec-53*, and Y51F10.7) and two genes (Y105C5A.13 and Y41C4A.11) that are induced during infection (Table S3). Relative transcript abundance was determined at 40 hpi, because this is a time point when we see large differences in spore levels. Here we found that *mdl-1* was not required for regulating gene expression upon infection (Figure 4). In particular, we found that the infection-repressed genes we tested are still repressed upon infection in *mdl-1* mutants, like in wild-type animals (Figure 4). Similarly, infection-induced genes are still induced in *mdl-1(tm311)* animals, like in wild-type animals (Figure 4). A gene known to be highly induced during infection, C17H1.6, was used a positive control for gene induction upon infection (Bakowski *et al.* 2014), and the increase in transcript was at least 1000-fold in infected animals (data not shown). We also analyzed whether *mdl-1* regulates expression of these genes in the absence of infection, and found that *mdl-1* appears to play a minor role in regulating basal expression of some of these genes (Figure 4). In summary, through querying a subset of infection response genes that had predicted MDL-1 binding sites, we did not find a role for MDL-1 in mediating the transcriptional response to infection.

### MDL-1::GFP and MML-1::GFP are expressed in the intestine during infection

**Figure 5:**
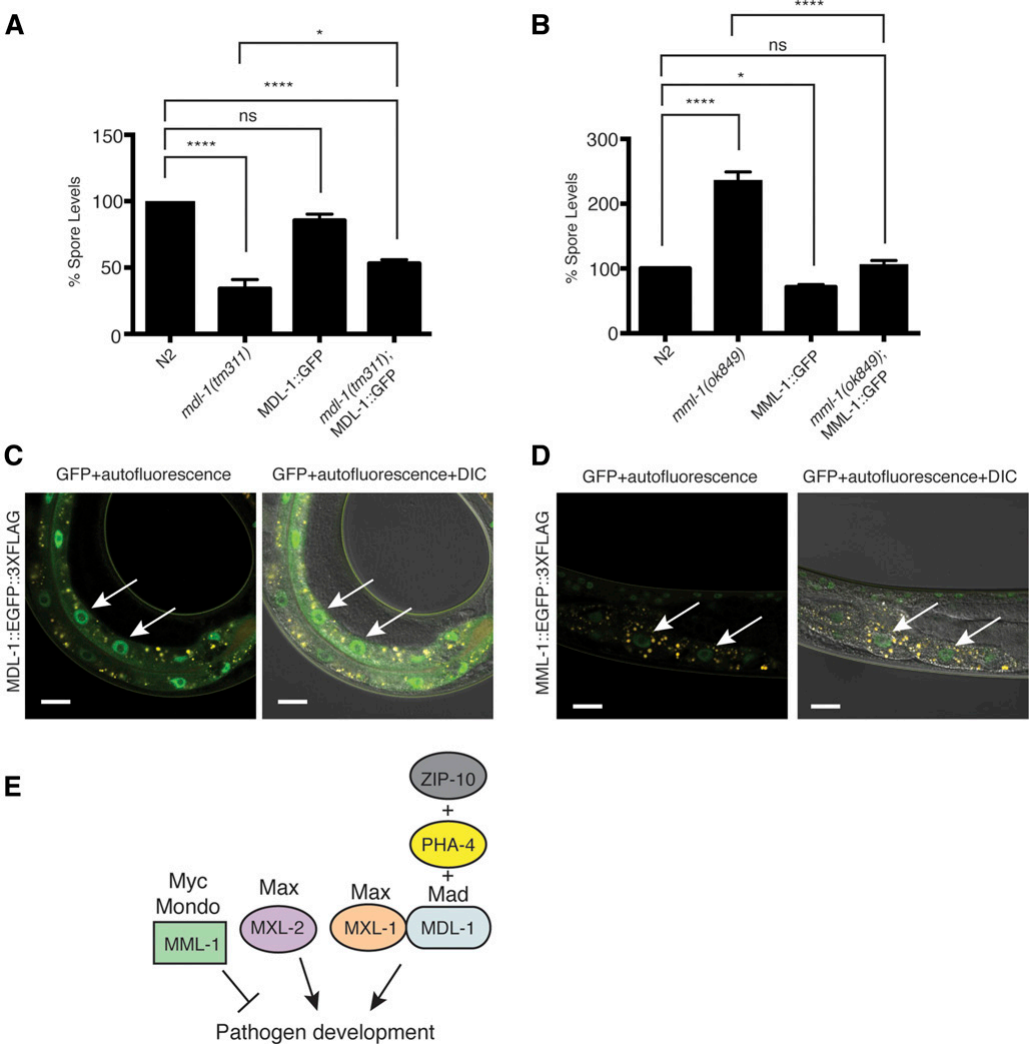
MDL-1::GFP-3×FLAG and MML-1::GFP-3×FLAG transgenes complement the mutant phenotypes and are expressed in the intestine during infection. (A) Spore levels of N2, *mdl-1(tm311)*, MDL-1::GFP::3×FLAG, and *mdl-1(tm311)*; MDL-1::GFP::3×FLAG *C. elegans*. (B) Spore level values of N2, *mml-1(ok849)*, MML-1:: GFP::3×FLAG, and *mml-1(ok849)*; MML-1::GFP::3×FLAG animals. For (A) and (B), spore levels are the average of three independent experiments; error bars represent SEM. ns, not significant. * *P* < 0.05, **** *P* < 0.0001. (C) Expression of MDL-1:: GFP::3×FLAG in animals 24 hpi with *N. parisii*. (D) Expression of MML-1::GFP::3×FLAG in animals 24 hpi with *N. parisii*. For (C) and (D), arrows indicate intestinal nuclei expressing GFP, GFP is in green, autofluorescence in yellow, and scale bars are 20 µm. Left image for each shows GFP+ autofluorescence and right images show GFP+ autofluorescence + DIC brightfield. (E) Model for noncanonical wiring of Myc interaction network during *N. parisii* infection of *C. elegans*, based on epistasis analysis. MDL-1 acts with PHA-4 and ZIP-10 to promote pathogen development. It also acts together with MXL-1, likely as a heterodimeric binding partner to promote pathogen development. In parallel MML-1 inhibits pathogen development, while MXL-2 promotes pathogen development.

To investigate the expression of MDL-1 and MML-1 proteins—two transcription factors with opposing and strong effects on spore levels—we analyzed animals that contain integrated versions of either MDL-1::GFP::3×FLAG or MML-1::GFP::3×FLAG transgenes. Both of these transgenes were generated by the TransgeneOme project, and consist of genomic DNA with a GFP::3×FLAG tag integrated at the C-terminus of each gene, flanked by several kilobases of regulatory region upstream and downstream of the gene of interest (Sarov *et al.* 2012). First we investigated whether these tagged versions of the transcription factors could complement the mutant phenotypes, which would indicate that they are functional, and give confidence that they reflect endogenous protein expression. Here, we found that there was partial complementation for the reduced spore levels phenotype of *mdl-1* mutants with the MDL-1::GFP::3×FLAG transgene (Figure 5A), and full complementation for the increased spore level phenotype of *mml-1(ok849)* mutants with MML-1::GFP::3×FLAG (Figure 5B). Next, we examined their expression by Western blots and with fluorescence imaging. Here we found that both the MDL-1::GFP::3×FLAG transgene, and the MML-1::GFP::3×FLAG transgenes produced proteins of the expected sizes on a Western blot (Figure S3). When we analyzed tissue distribution of these transgenic proteins, we found that both GFP-tagged proteins were expressed in intestinal nuclei during infection (Figure 5, C and D). Altogether, these data suggest that MML-1 and MDL-1 are likely expressed during infection within intestinal cells, the site of infection by *N. parisii*.

## Discussion

We have described an RNAi-based genetic screen to identify host transcription factors that are important for growth and development of the microsporidian pathogen *N. parisii* in its natural host, *C. elegans*. Despite the Microsporidia phylum containing over 1400 species, prior to this study almost nothing was known about the host factors important for the intracellular development of any species of microsporidia. Using our *N. parisii*/*C. elegans* screening strategy, we identified nine host genes whose knockdown decreases spore levels, and through investigating the Myc pathway further, identified a gene whose knockdown increases spore levels. These genes represent the first description of host transcription factors that regulate microsporidia growth and development. Because we screened a library of transcription factors, it is likely that these host genes are regulators of various processes (metabolism, stress response, etc.) that may be important for different aspects of pathogen growth, including production of essential metabolites, or helping the host cells cope with the incredibly rapid replication of the pathogen within the host cell. Further analysis of these transcription factors, including identifying potential downstream pathways and effectors that contribute to the observed spore-levels phenotypes will be critical for understanding microsporidian success as an intracellular pathogen.

Some of the major hits from our screen for host factors that regulate pathogen development are players in the Myc interaction network of transcription factors. In mammals, this network contains 11 transcription factors: three Myc, four Mad/Mxi, two Mnt/Mga, and two Mondo proteins, and these transcription factors are important regulators of cell growth, proliferation, and energy metabolism. Interestingly, two intracellular apicomplexan parasites, *Toxoplasma gondii* and *Theleria*, have been shown to upregulate mammalian c-Myc upon infection (Franco *et al.* 2014; Dessauge *et al.* 2005), so mammals and nematodes may share a role for Myc interaction network factors in the response to intracellular parasites. However, the connection between these findings is not clear, given that *C. elegans* lacks a clear Myc ortholog. Further analysis of mammalian Myc interaction network players that have clear *C. elegans* orthologs could shed light on this question. The *C. elegans* Myc interaction network is highly reduced, being comprised of only four major players, *mdl-1*, *mxl-1*, *mxl-2*, and *mml-1*, with MDL-1:MXL-1 (Mad-Max) heterodimers and MML-1:MXL-2 (Myc-Mondo-Max) heterodimers. Of note, a previous study that indicated these two heterodimers have opposing functions in lifespan also indicated that they regulate expression of genes that are associated with pathogen response and immunity, but the significance of this regulation is unknown (Johnson *et al.* 2014).

In this work we have shown that *mdl-1* and *mml-1* have strong and opposing effects on the growth and development of microsporidia within *C. elegans* intestinal cells. MDL-1 appears to be acting canonically with MXL-1 to promote pathogen growth. In contrast, MML-1 appears to inhibit pathogen growth, and be acting independently of its standard binding partner, MXL-2, which promotes pathogen growth. Thus, it may be that MML-1 acts with a different heterodimeric binding partner to inhibit pathogen development, and similarly MXL-2 acts with a different heterodimeric binding partner to promote pathogen development (Figure 5E). *C. elegans* has a paralog of MXL-1, called MXL-3, which regulates lipolysis and autophagy in response to nutrient availability (O’Rourke and Ruvkun 2013), and can also interact with the transcription factor SKN-1 to mediate a starvation response (Paek *et al.* 2012), but this protein is not thought to interact with other members of the Myc interaction network of transcription factors (Grove *et al.* 2009). Future work could investigate the role of MXL-3 in regulating spore levels.

Recent findings by Nakamura *et al.* (2016) have shown that MML-1 and MXL-2 are responsive to signals from the germline to promote increased lifespan. Transcriptional profiling of *mml-1* and *mxl-2* mutants revealed that these transcription factors regulate an overlapping cohort of target genes, although MXL-2 appears to regulate a large number of genes independently of MML-1. Thus MXL-2 is likely interacting with other binding partners to regulate gene expression. Given that our study indicates that MML-1 and MXL-2 have distinct functions in regulating pathogen development, interaction between MXL-2 and other transcription factors may be a common mechanism to achieve distinct outputs by this factor. Another important finding from Nakamura *et al.* (2016) is that MML-1 and MXL-2 signaling leads to an upregulation of autophagy gene expression. Interestingly, ubiquitin-mediated responses and autophagy have previously been shown to provide defense against *N. parisii* in *C. elegans*, although *N. parisii* infection does not upregulate transcription of autophagy genes directly, but rather upregulates expression of ubiquitin ligase components that could act upstream of autophagy (Bakowski *et al.* 2014). Nakamura *et al.* (2016) showed that the MML-1/MXL-2 regulation of autophagy is found only in animals lacking a germline, such as the *glp-1(e2141)* mutants that we used in our RNAi screen. However, in our study, the effect of MXL-2 on spore levels was the same whether animals have a germline or not. Thus, it is unlikely that the effect of MXL-2 and MML-1 on pathogen development is due solely to their regulation of autophagy via germline removal as described by Nakamura *et al.* (2016).

We also identified other transcription factors from our screen for regulators of microsporidia growth, and found that two of them appear to act together with MDL-1 based on epistasis analysis (Figure 5E). One of them, *pha-4*, is a transcription factor whose putative target genes largely overlap with those of *mdl-1* (Johnson *et al.* 2014), and we found that it has no additive effect on spore levels when it is mutated together with *mdl-1*. Analysis of these two factors for their effects on lifespan showed similar interactions (Johnson *et al.* 2014). We also identified a novel genetic interaction between the bZIP transcription factor *zip-10* and *mdl-1*. *zip-10* has previously been described to regulate *C. elegans* body size and male tail morphogenesis downstream of the TGF-β signaling ligand *dbl-1*, and the smad transcription factor *sma-9* (Liang *et al.* 2007). However, little is known about the effectors downstream of *zip-10*, particularly within intestinal cells, which is the site of *N. parisii* infection. MDL-1 does not appear to regulate the transcriptional response to infection, but there are likely to be multiple effectors downstream of MDL-1 and ZIP-10, as well as downstream of MXL-1 and MXL-2, that are promoting intracellular pathogen development, while effectors downstream of MML-1 likely inhibit intracellular pathogen development. Further analysis of the interaction of *zip-10* and *mdl-1* both genetically and biochemically, including understanding the upstream signaling that regulate these transcription factors, and downstream effectors of these transcription factors will enhance our understanding of how an intracellular pathogen hijacks host resources, and also provide new understanding into the plasticity of the Myc interaction network in *C. elegans*.

## Supplementary Material

Supplemental Material
