# Supplementary material for: Microsporidia Intracellular Development Relies on Myc Interaction Network Transcription Factors in the Host
Source: G3 (Bethesda). 2016 Jul 5;6(9):2707–16. doi: 10.1534/g3.116.029983 (PMC5015929; doi:10.1534/g3.116.029983)
Supplement: Supplemental Material [file supp_6_9_2707__index.html]

Microsporidia Intracellular Development Relies on Myc Interaction Network Transcription Factors in the Host — Supplemental Material 

# Microsporidia Intracellular Development Relies on Myc Interaction Network Transcription Factors in the Host

## Supplemental Material for Botts, *et al*, 2016

**Files in this Data Supplement:**

- Figure S1 - DY96 staining of spores inside infected *C. elegans*. (.ai, 2.7 MB)
- Figure S2 - Quantifying *N. parisii* sporoplasms in intestinal cells as pathogen invasion events. (.tif, 1.3 MB)
- Figure S3 - Western blot and qRT-PCR analysis of MDL-1 and MML-1 transgene expression and comparison to endogenous mRNA expression. (.tif, 1.6 MB)
- Table S1 - Mean DY96 signals for RNAi clones screened. (.xlsx, 77 KB)
- Table S2 - Contingency table for transcription factor binding sites and enrichment in infection-regulated genes. (.xlsx, 18 KB)
- Table S3 - RNAseq analysis from Bakowski et al. for genes used for qRT-PCR analysis. (.xlsx, 45 KB)
