# Supplementary figures and images for: Microsporidia Intracellular Development Relies on Myc Interaction Network Transcription Factors in the Host
Source: G3 (Bethesda). 2016 Jul 5;6(9):2707–16. doi: 10.1534/g3.116.029983 (PMC5015929; doi:10.1534/g3.116.029983)

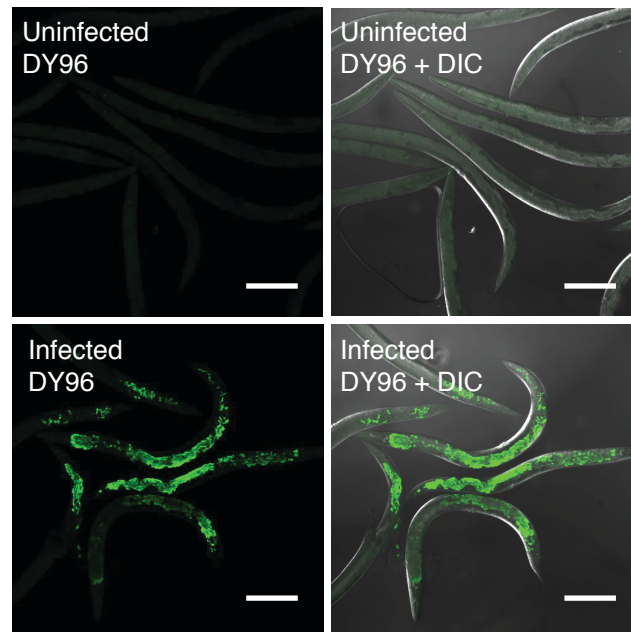

Figure S1

Supplement: Supplemental Material [file supp_g3.116.029983_FigureS1.ps]

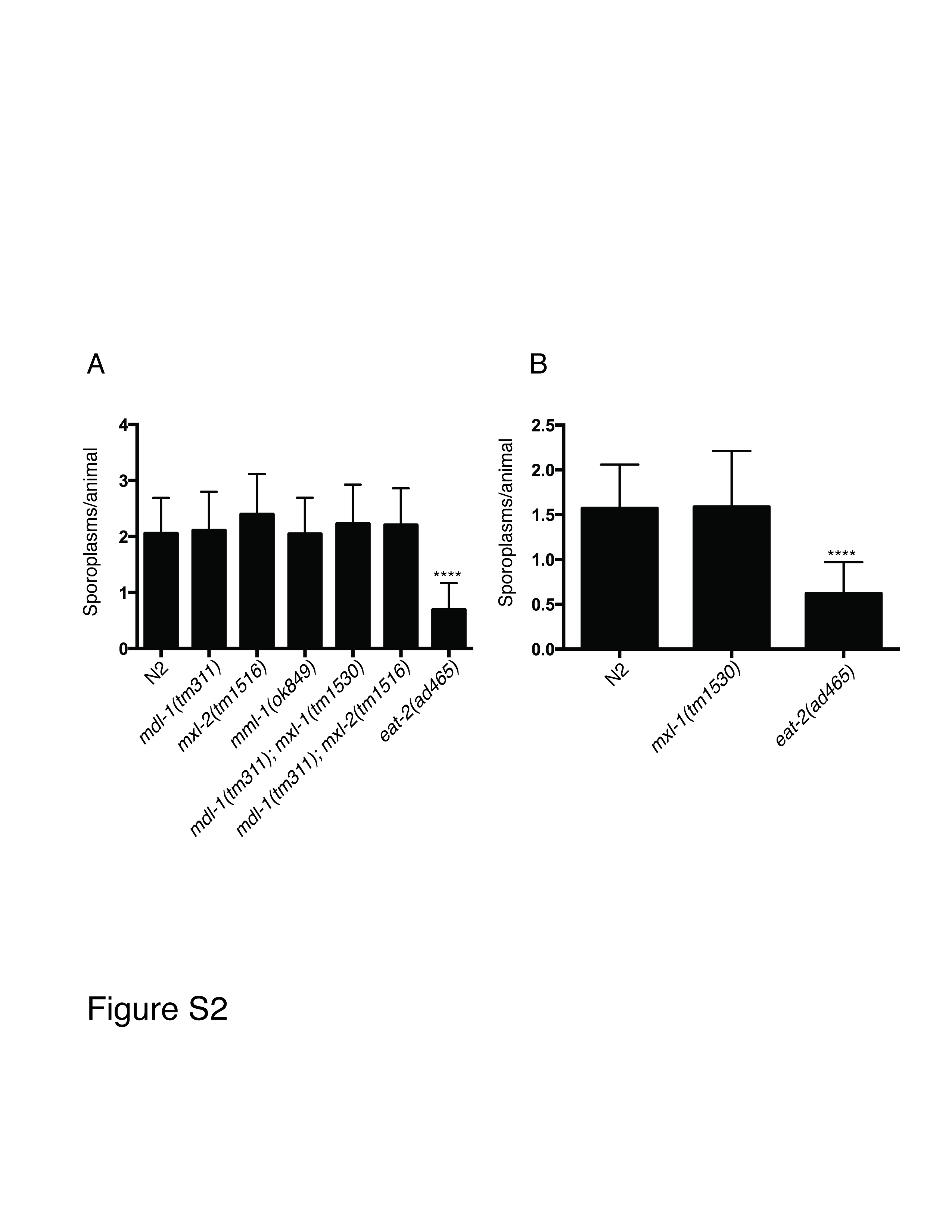

Supplement: Supplemental Material [file supp_g3.116.029983_FigureS2.tif]

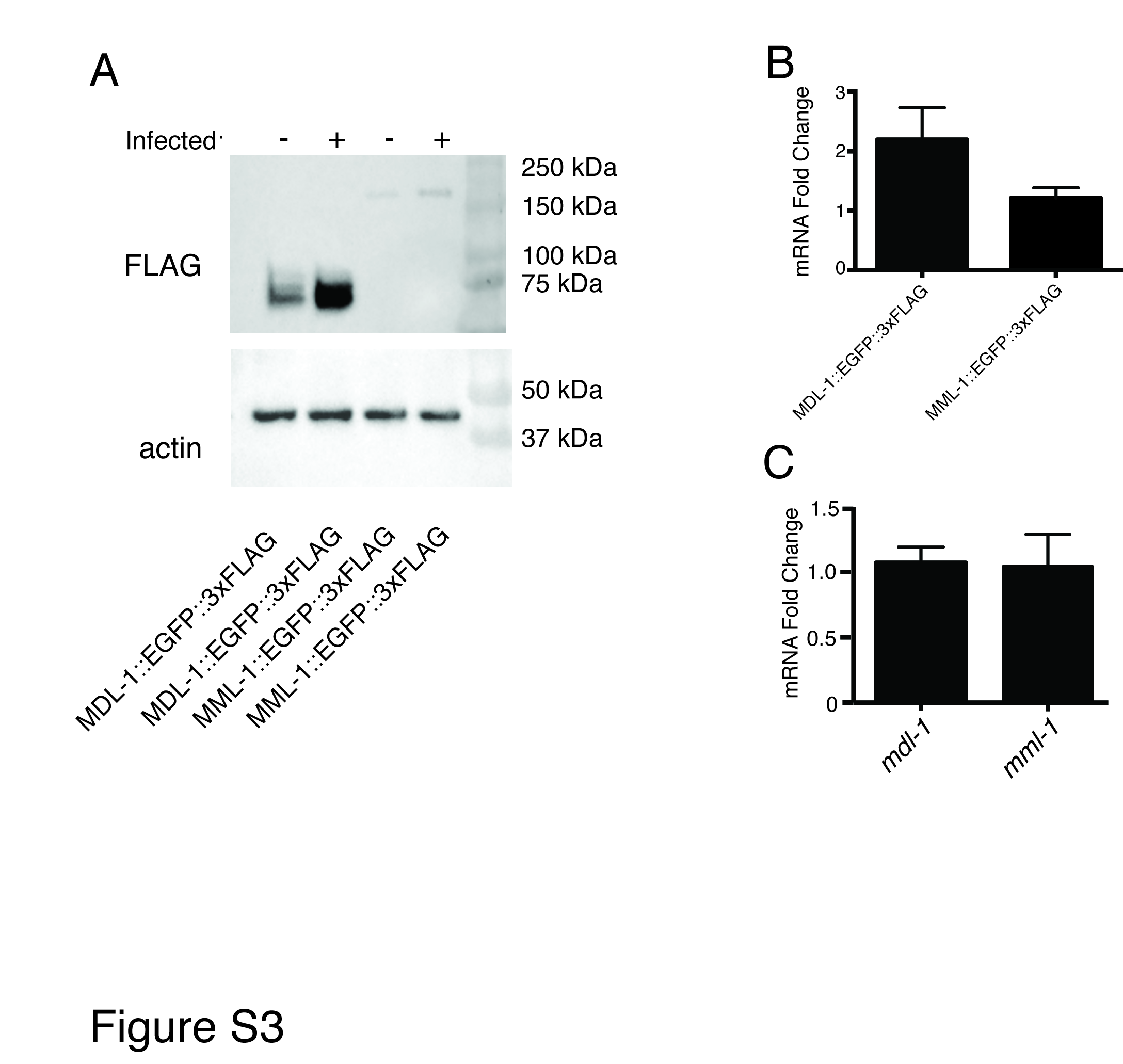

Supplement: Supplemental Material [file supp_g3.116.029983_FigureS3.tif]
